# Latent Tuberculosis Infection and Associated Factors in Patients with Systemic Lupus Erythematosus: a Multicenter, Cross-Sectional Study

**DOI:** 10.1128/spectrum.00848-23

**Published:** 2023-05-09

**Authors:** Lifan Zhang, Yanan Ma, Nan Jiang, Xiaoqing Zou, Yueqiu Zhang, Fengchun Zhang, Xiaofeng Zeng, Yan Zhao, Shengyun Liu, Xiaoxia Zuo, Huaxiang Wu, Lijun Wu, Hongbin Li, Zhiyi Zhang, Sheng Chen, Ping Zhu, Miaojia Zhang, Wencheng Qi, Yi Liu, Huaxiang Liu, Xiaochun Shi, Xiaoqing Liu

**Affiliations:** a Division of Infectious Diseases, Department of Internal Medicine, State Key Laboratory of Complex Severe and Rare Disease, Peking Union Medical College Hospital, Chinese Academy of Medical Sciences and Peking Union Medical College, Beijing, People’s Republic of China; b Centre for Tuberculosis Research, Chinese Academy of Medical Sciences and Peking Union Medical College, Beijing, People’s Republic of China; c Clinical Epidemiology Unit, International Epidemiology Network, Peking Union Medical College Hospital, Chinese Academy of Medical Sciences, Beijing, People’s Republic of China; d 4+4 Medical Doctor Program, Chinese Academy of Medical Sciences and Peking Union Medical College, Beijing, People’s Republic of China; e School of Population Medicine and Public Health, Chinese Academy of Medical Sciences and Peking Union Medical College, Beijing, People’s Republic of China; f Department of Rheumatology and Clinical Immunology, Peking Union Medical College Hospital, Chinese Academy of Medical Sciences and Peking Union Medical College, Key Laboratory of Rheumatology and Clinical Immunology, Ministry of Education, Beijing, People’s Republic of China; g Department of Rheumatology and Immunology, First Affiliated Hospital of Zhengzhou University, Zhengzhou, People’s Republic of China; h Department of Rheumatology and Immunology, Xiangya Hospital, Central South University, Changsha, People’s Republic of China; i Department of Rheumatology, Second Affiliated Hospital of Zhejiang University School of Medicine, Hangzhou, People’s Republic of China; j Department of Rheumatology, People’s Hospital of Xinjiang Uygur Autonomous Region, Urumchi, People’s Republic of China; k Department of Rheumatology and Immunology, Affiliated Hospital of Inner Mongolia Medical University, Hohhot, People’s Republic of China; l Department of Rheumatology and Immunology, First Affiliated Hospital of Harbin Medical University, Harbin, People’s Republic of China; m Department of Rheumatology, Renji Hospital, School of Medicine, Shanghai Jiao Tong University, Shanghai, People’s Republic of China; n Department of Clinical Immunology, Xijing Hospital, Fourth Military Medical University, Xi’an, People’s Republic of China; o Department of Rheumatology, First Affiliated Hospital of Nanjing Medical University, Nanjing, People’s Republic of China; p Department of Rheumatology, Tianjin First Central Hospital, Tianjin, People’s Republic of China; q Department of Rheumatology and Immunology, West China Hospital, Sichuan University, Chengdu, People’s Republic of China; r Department of Rheumatology, Qilu Hospital of Shandong University, Ji’nan, People’s Republic of China; Quest Diagnostics Nichols Institute

**Keywords:** latent tuberculosis infection, systemic lupus erythematosus, China, T-SPOT.TB

## Abstract

The objectives of this study were to screen for latent tuberculosis infection (LTBI) among patients with systemic lupus erythematosus (SLE) using the T-SPOT.TB assay and to identify factors affecting the assay results. SLE patients were enrolled from 13 tertiary hospitals in eastern, central, and western China from September 2014 to March 2016 and were screened using the T-SPOT.TB assay to detect LTBI. Basic information about the subjects was collected, including gender, age, body mass index (BMI), course of disease, evidence of previous tuberculosis, Systemic Lupus Erythematosus Disease Activity Index 2000 (SLEDAI-2K) score, and the use of glucocorticoids and immunosuppressants. Univariate analysis and multivariable logistic regression were performed to identify factors affecting the results of the T-SPOT.TB assay. In all, 2,229 SLE patients were screened using the T-SPOT.TB assay, of whom 334 patients tested positive, yielding a positivity rate of 15% (95% confidence interval [CI], 13.5% to 16.5%). The positivity rate was higher in male than female patients and had an increasing trend with age. Multivariable logistic regression analysis showed that patients over 40 (odds ratio [OR], 1.65; 95% CI, 1.29 to 2.10) and with evidence of previous tuberculosis (OR, 4.43; 95% CI, 2.81 to 6.99) were more likely to have positive T-SPOT.TB results, while patients with a SLEDAI-2K score of ≥10 (OR, 0.61; 95% CI, 0.43 to 0.88), a glucocorticoid dose of ≥60 mg/d (OR, 0.62; 95% CI, 0.39 to 0.98), leflunomide (LEF) treatment (OR, 0.51; 95% CI, 0.29 to 0.88), or tacrolimus (FK506) treatment (OR, 0.40; 95% CI, 0.16 to 1.00) were more likely to have negative T-SPOT.TB results. The frequencies of CFP-10–specific gamma interferon (IFN-γ)-secreting T cells were significantly lower in SLE patients with severe disease activity or high-dose glucocorticoids (*P* < 0.05). The positivity rate of the T-SPOT.TB assay was 15% among SLE patients. Severe, active SLE disease and the use of high-dose glucocorticoids and some types of immunosuppressants are likely to result in negative T-SPOT.TB results. For SLE patients with the above conditions, diagnosing LTBI based on a positive T-SPOT.TB result may lead to underestimation of the prevalence.

**IMPORTANCE** The burden of tuberculosis and systemic lupus erythematosus in China ranks among the top three in the world. Therefore, active screening for LTBI and preventive intervention in SLE patients are of great significance in China. In view of the lack of relevant data in a large sample, we conducted a multicenter, cross-sectional study using T-SPOT.TB as a screening method for LTBI, to investigate the prevalence of LTBI and analyze the factors affecting the results of the T-SPOT.TB assay in SLE patients. Our study showed that the overall positivity rate of the T-SPOT.TB assay in SLE patients was 15.0%, which was lower than the estimated LTBI prevalence in the general population in China (~20%). For SLE patients with severe, active disease, high-dose glucocorticoids, and some types of immunosuppressants, a diagnosis of LTBI based on only positive T-SPOT.TB results may lead to underestimation of the prevalence.

## INTRODUCTION

Prior to coronavirus disease 2019 (COVID-19), tuberculosis led to the highest number of deaths among all infectious diseases. China was estimated to have 842,000 new tuberculosis cases in 2020, second only to India (1). Systemic lupus erythematosus (SLE) is a rheumatic immune disease with the most complex pathogenesis, the most prominent multi-organ involvement, the strongest clinical heterogeneity, and the highest proportion of patients (2). In 2020, China had approximately 1 million SLE patients, with an estimated prevalence of 30/100,000 to 70/100,000, ranking second in the world (3).

About 90% of people infected with Mycobacterium tuberculosis do not develop active tuberculosis (ATB) directly but are in a state of latent tuberculosis infection (LTBI) without clinical symptoms. The LTBI prevalence was estimated to be 20.3% among people aged 15 years and above in China, and there were nearly 300 million LTBI (4). LTBI has a 5% to 10% risk of progressing to ATB in the future, becoming a new source of infection. The risk of ATB in SLE patients was found to be 3 to 10 times that of the general population due to immune dysfunction caused by the disease itself, as well as by the use of glucocorticoids and immunosuppressants (5). The clinical symptoms are often atypical when SLE is complicated with ATB. Misdiagnosis is common because it is difficult to distinguish ATB from the activity of SLE or other infections. Moreover, such patients tend to be severely ill and have a high mortality rate.

Therefore, active screening and preventive intervention for LTBI in SLE patients will effectively reduce the incidence rate, mortality rate, and transmission risk of ATB, as well as medical costs, bringing about significant health and social benefits. Currently, there is no gold standard for LTBI diagnosis. Two internationally recognized detection methods are the tuberculin skin test (TST) and interferon gamma release assays (IGRAs), both of which measure cellular immune responses. TST may be falsely positive due to BCG vaccination and nontuberculous *Mycobacteria* (NTM) infection, and it has low sensitivity in an immunocompromised population (6). In comparison, IGRAs are a better tool for identifying LTBI in such a population, including SLE patients, and T-SPOT.TB, based on the enzyme-linked immunospot (ELISPOT) assay, in particular has a higher positive rate (7). In 2022, China established an expert consensus for the diagnosis and management of LTBI in patients with rheumatic diseases. The IGRA is recommended for use as the preferred method for screening for LTBI in patients with rheumatic diseases, due to its higher accuracy compared to the TST (8). Until now, research on the prevalence of LTBI in patients with rheumatic immune diseases has mainly focused on arthritis patients treated with tumor necrosis factor alpha (TNF-α) antagonists (9, 10), while research on the prevalence of LTBI in SLE patients has lacked multicenter, large-sample data for all countries.

This study is a multicenter cross-sectional study based on tertiary hospitals in China, using T-SPOT.TB as a screening method for LTBI. The prevalence of LTBI in SLE patients was investigated, and factors affecting the results of T-SPOT.TB were analyzed.

## RESULTS

### Basic situation.

The ETHERTB study enrolled a total of 2,918 SLE patients without ATB, 2,229 (76.4%) of whom underwent testing using the T-SPOT.TB assay. Basic information of the two groups of SLE patients is shown in Table 1.

**TABLE 1:** Basic information of SLE patients with and without T-SPOT.TB assay

Characteristic	Data for SLE patients		*P* value
	With T-SPOT.TB	Without T-SPOT.TB	
Total (*n*)	2,229	689	
Gender (*n* [%])			
Male	164 (7.4)	53 (7.7)	0.770
Female	2,065 (92.6)	636 (92.3)	
Mean age ± SD (yrs)	37 ± 12	38 ± 13	0.082
16–40 (*n*)	1,411	413	0.111
>40 (*n*)	818	276	
BMI (Kg/m^2^[Mean ± SD])	22.4 ± 3.7	22.4 ± 3.6	0.984
Median course of disease (mo [IQR])^*a*^	23 (3–63)	27 (2–67)	0.653
Evidence of previous tuberculosis (*n* [%])	86 (3.9)	19 (2.8)	0.185
Median SLEDAI (IQR)	5 (2–10)	6 (2–10)	0.536
0–4 (*n*)	1,113	318	0.152
5–9 (*n*)	540	189	
≥10 (*n*)	576	182	
Dose of glucocorticoids (mg/d [median {IQR}])	15 (7.5–47.5)	15 (5–50)	0.219
Immunosuppressants (*n* [%])^*b*^			
CTX	360 (16.2)	136 (19.7)	0.028
MMF	442 (19.8)	123 (17.9)	0.251
MTX	137 (6.1)	51 (7.4)	0.241
AZA	53 (2.4)	31 (4.5)	0.004
LEF	150 (6.7)	51 (7.4)	0.542
CsA	65 (2.9)	16 (2.3)	0.407
FK506	83 (3.7)	16 (2.3)	0.076

a IQR, interquartile range.

b CTX, cyclophosphamide; MMF, mycophenolate mofetil; MTX, methotrexate; AZA, azathioprine; LEF, leflunomide; CsA, cyclosporine; FK506, tacrolimus.

### Prevalence of LTBI in SLE patients.

In this study, no indeterminate T-SPOT.TB results were observed. In total, 2,229 SLE patients had definitive T-SPOT.TB results, 334 of which were positive. The positive rate of T-SPOT.TB in SLE patients was 15.0% (95% confidence interval [CI], 13.5 to 16.5). Male patients had a higher positive rate of T-SPOT.TB compared to female patients, and the positivity rate increased with age (*P* < 0.001) (Table 2).

**TABLE 2:** Prevalence of LTBI among SLE patients

Characteristic	No. of SLE patients:		Prevalence of LTBI (% [95% CI])	*P* value
	With positive T-SPOT.TB result	Total		
Total	334	2,229	15.0 (13.5–16.5)	
Gender				0.304
Male	27	164	16.5 (11.1–23.0)	
Female	307	2,065	14.9 (13.4–16.5)	
Age (yrs)				<0.001
≤20	5	116	4.3 (1.4–9.8)	
21–30	76	703	10.8 (8.6–13.3)	
31–40	89	592	15.0 (12.3–18.2)	
41–50	96	493	19.5 (16.1–23.2)	
51–60	48	225	21.3 (16.2–27.3)	
>60	20	100	20.0 (12.7–29.2)	

### Univariate analysis of potential factors affecting the results of T-SPOT.TB in SLE patients.

SLE patients aged over 40 years old (odds ratio [OR], 1.83; 95% CI, 1.45 to 2.31) and those who had evidence of previous tuberculosis (OR, 5.20; 95% CI, 3.34 to 8.09) were more likely to have positive T-SPOT.TB results. Moderate (OR, 0.69; 95% CI, 0.52 to 0.93) to severe (OR, 0.48; 95% CI, 0.35 to 0.65) disease activity, moderate (OR, 0.68; 95% CI, 0.50 to 0.91) to high (OR, 0.46; 95% CI, 0.30 to 0.69) dose of glucocorticoids, and treatment with leflunomide (LEF) (OR, 0.61; 95% CI, 0.36 to 1.06) or tacrolimus (FK506) (OR, 0.35; 95% CI, 0.14 to 0.88) were associated with negative T-SPOT.TB results (Table 3).

**TABLE 3:** Univariate analysis of potential factors affecting the results of T-SPOT.TB in SLE patients

Characteristic	No. of SLE patients with:		Univariate analysis	
	Positive T-SPOT.TB results	Negative T-SPOT.TB results		
			OR (95% CI)	*P* value
Total	334	1,895		
Gender				
Male	27	137	1	
Female	307	1,758	0.89 (0.58–1.36)	0.582
Age (yrs)				
16–40	170	1,241	1	
>40	164	654	1.83 (1.45–2.31)	<0.001
BMI (Kg/m^2^[Mean ± SD])	22.5 ± 6.1	22.5 ± 3.6	1.00 (0.98–1.02)	0.940
Median course of disease (mo [IQR])	23 (3–63)	25 (5–67)	1.00 (1.00–1.00)	0.524
Evidence of previous tuberculosis				
No	295	1,848	1	
Yes	39	47	5.20 (3.34–8.09)	<0.001
SLEDAI-2K score				
0–4	205	908	1	
5–9	73	467	0.69 (0.52–0.93)	0.013
≥10	56	520	0.48 (0.35–0.65)	<0.001
Dose of glucocorticoids (mg/d)				
0–29	242	1,153	1	
30–59	64	490	0.68 (0.50–0.91)	0.010
≥60	28	252	0.46 (0.30–0.69)	<0.001
Immunosuppressants^*a*^				
CTX				
No	287	1,582	1	
Yes	47	313	0.83 (0.59–1.15)	0.263
MMF				
No	261	1,526	1	
Yes	73	369	1.16 (0.87–1.54)	0.314
MTX				
No	316	1,776	1	
Yes	18	119	0.85 (0.51–1.42)	0.533
AZA				
No	325	1,851	1	
Yes	9	44	1.17 (0.56–2.41)	0.680
LEF				
No	319	1,760	1	
Yes	15	135	0.61 (0.36–1.06)	0.079
CsA				
No	322	1,842	1	
Yes	12	53	1.30 (0.69–2.45)	0.427
FK506				
No	329	1,817	1	
Yes	5	78	0.35 (0.14–0.88)	0.026

a CTX, cyclophosphamide; MMF, mycophenolate mofetil; MTX, methotrexate; AZA, azathioprine; LEF, leflunomide; CsA, cyclosporine; FK506, tacrolimus.

### Multivariable logistic regression analysis of factors affecting the results of T-SPOT.TB in SLE patients.

SLE patients aged over 40 years old (OR, 1.65; 95% CI, 1.29 to 2.10) and those who had evidence of previous tuberculosis (OR, 4.43; 95% CI, 2.81 to 6.99) were more likely to have positive T-SPOT.TB results. Severe disease activity (OR, 0.61; 95% CI, 0.43 to 0.88), high-dose glucocorticoids (OR, 0.62; 95% CI, 0.39 to 0.98), LEF (OR, 0.51; 95% CI, 0.29 to 0.88), and FK506 (OR, 0.40; 95% CI, 0.16 to 1.00) were associated with negative T-SPOT.TB results (Table 4). For SLE patients with no use of LEF or FK506, a Systemic Lupus Erythematosus Disease Activity Index 2000 (SLEDAI-2K) score of ≤4, and a glucocorticoid dose of <30 mg/d, the positivity rate of the T-SPOT.TB assay was 20.6% (173/840); for patients satisfying the above conditions but with no use of glucocorticoids, the positivity rate of T-SPOT.TB was 24.5% (12/49).

**TABLE 4:** Multivariable logistic regression analysis of factors affecting the results of T-SPOT.TB in SLE patients

Characteristic	No. of SLE patients with:		Multivariate analysis	
	Positive T-SPOT.TB results	Negative T-SPOT.TB results
			OR (95% CI)	*P* value
Total	334	1,895		
Age (yrs)				
16–40	170	1,241	1	
>40	164	654	1.65 (1.29–2.10)	<0.001
Evidence of previous tuberculosis				
No	295	1,848	1	
Yes	39	47	4.43 (2.81–6.99)	<0.001
SLEDAI-2K				
0–4	205	908	1	
5–9	73	467	0.79 (0.58–1.08)	0.145
≥10	506	520	0.61 (0.43–0.88)	0.008
Dose of glucocorticoids (mg/d)				
0–29	242	1,153	1	
30–59	64	490	0.81 (0.58–1.12)	0.195
≥60	28	252	0.62 (0.39–0.98)	0.041
Immunosuppressants^*a*^				
LEF				
No	319	1,760	1	
Yes	15	135	0.51 (0.29–0.88)	0.017
FK506				
No	329	1,817	1	
Yes	5	78	0.40 (0.16–1.00)	0.05

a LEF, leflunomide; FK506, tacrolimus.

### Frequencies of M. tuberculosis-specific IFN-γ secreting T cells.

**FIG 1:**
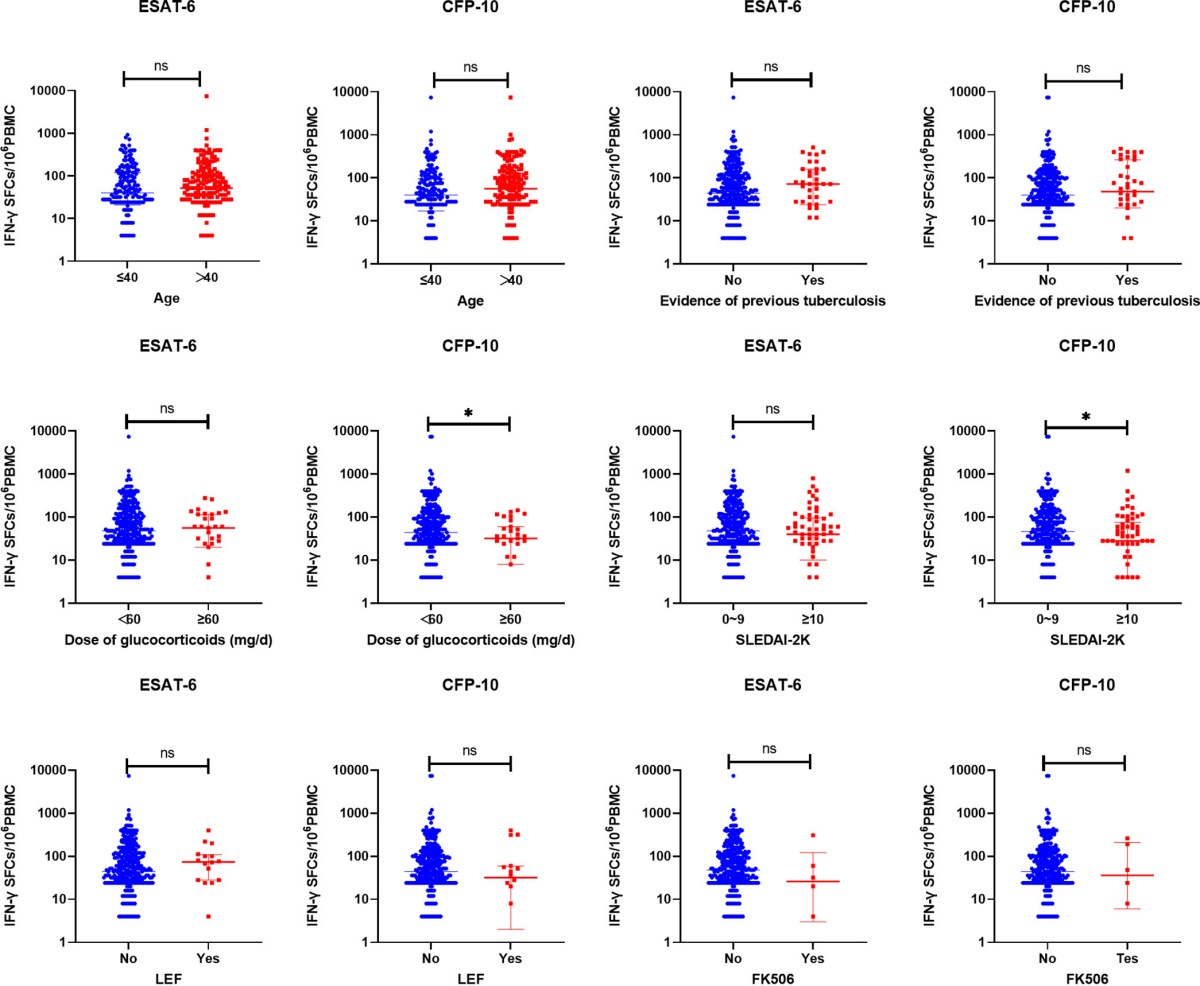
Frequencies of ESAT-6- and CFP-10-specific IFN-γ–secreting T cells between different immunity states (*, *P* < 0.05; ns, no significance). PBMC, peripheral blood mononuclear cell; LEF, leflunomide; FK506, tacrolimus; SFCs, Spots forming cells.

Based on the multivariable analysis, further analysis on the frequencies of M. tuberculosis-specific gamma interferon (IFN-γ)-secreting T cells was conducted. The results showed that the frequencies of CFP-10–specific IFN-γ–secreting T cells were significantly lower in SLE patients with severe disease activity or high-dose glucocorticoids (*P* < 0.05). No significant differences were observed in the frequencies of ESAT-6–specific IFN-γ–secreting T cells among patients with different immune status (Fig. 1; see Table S1 in the supplemental material).

## DISCUSSION

According to the fifth national epidemiological survey in China, the prevalence of ATB in the general population was 459/100,000 (11). Our previous single-center retrospective study found that the prevalence of ATB in hospitalized SLE patients was as high as 2,400/100,000, which was 6 times that of the general population (12). Compared with patients with ATB alone, the proportion of extrapulmonary tuberculosis is higher in SLE patients complicated with ATB, as is the proportion of severe tuberculosis, such as hematogenous disseminated pulmonary tuberculosis and central nervous system tuberculosis. A recent meta-analysis by Q. Wu et al. integrating 35 studies in 13 countries estimated the prevalence of ATB in SLE patients to be as high as 35.90/100,000 (13).

LTBI patients are a huge reservoir of ATB, and its management is essential for TB control. Given the vast number of LTBI cases in China, prevention strategies for all LTBI patients can be challenging to implement. However, targeting high-risk LTBI individuals for early intervention remains a practical approach to prevention. Patients with rheumatic diseases, such as SLE, are at a higher risk for developing active TB. Therefore, LTBI screening and preventive treatment for SLE patients are effective means to reduce the incidence of tuberculosis. However, there is currently no gold standard for the diagnosis of LTBI. Focusing on patients with rheumatic immune diseases, a prospective study conducted by C.M.F. Gomes et al. compared the sensitivity of TST and IGRAs in diagnosing LTBI in arthritis patients treated with TNF-α antagonists and found that IGRAs could improve the diagnostic accuracy of LTBI compared with TST; T-SPOT.TB in particular had a higher positive rate (14). Mínguez et al. and Vassilopoulos et al. obtained consistent results in their studies (15, 16). A meta-analysis including 40 studies based on populations at high risk of tuberculosis (50,592 subjects) found that IGRA-positive individuals had a higher risk of developing ATB than TST-positive ones, and the former benefited more from preventive treatment (17). Another meta-analysis also found that positive IGRAs had greater predictive power for ATB than TST (18).

In this study, a positive T-SPOT.TB assay was used to diagnose LTBI, and the positive rate of T-SPOT.TB, or the prevalence of SLE-related LTBI, in SLE patients nationwide was 15.0%. The positivity rate was higher in male patients than in female patients and had an increasing trend with age. Studies based on multicenter survey data and spatial statistical modeling by L. Gao et al. estimated that the prevalence of LTBI in Chinese people aged 15 and over was 20.34%, showed an increasing trend with age, and was higher in male participants than in female participants (19, 20). The prevalence of LTBI in SLE patients in this study showed consistent trends with gender and age, but the overall prevalence was unexpectedly lower than the previous national estimate.

In view of the low LTBI prevalence, we performed univariate and multivariable analyses on factors affecting the results of the T-SPOT.TB assay and found that for SLE patients, severe disease activity, high-dose glucocorticoids, LEF, and FK506 were associated with negative T-SPOT.TB results. In patients with almost inactive SLE (SLEDAI score, ≤4), low-dose glucocorticoids (<30 mg/d), and no LEF or FK506 at baseline, the positivity rate of T-SPOT.TB in SLE patients was 20.6%, close to the national estimate of LTBI. Patients not using glucocorticoids were further screened; that is, in the case of even more stable SLE, the positivity rate of T-SPOT.TB was 24.5%, higher than the national estimate of LTBI.

Furthermore, we analyzed the frequencies of M. tuberculosis-specific IFN-γ–secreting T cells. Notably, our results indicated a significant decrease (*P* < 0.05) in the frequencies of CFP-10–specific IFN-γ–secreting T cells with increasing disease activity and increasing dose of glucocorticoids. This suggested that the immune responses mediated by CFP-10 might be more susceptible to the individual’s immune status. This result was consistent with the research findings of S. Liu et al. (21), but further studies with larger sample sizes are needed to support it. These findings all imply that the overall prevalence of LTBI in SLE patients may be underestimated using the current detection methods due to the influence of disease conditions and medications.

SLE is a chronic autoimmune disease characterized by abnormal T-cell immune responses. Excessive exposure of autoantigens in active SLE may cause T-cell exhaustion, resulting in suppressed Th1 signaling and a decreased level of IFN-γ (22). Sung et al. found that the IFN-γ level in active SLE patients was significantly lower than that of inactive SLE patients (23). When IFN-γ production is hampered by defective T cells, IGRAs may be less sensitive and generate false-negative results.

In addition to disease activity, the use of glucocorticoids and immunosuppressants can also affect T-cell responses through multiple mechanisms, thereby reducing the sensitivity of IGRAs. Studies found that glucocorticoids had significant negative impacts on both IGRAs and TST, and the impact on TST was greater (24). M. Aewnas et al. also found that in SLE patients, receiving glucocorticoids and/or other immunosuppressants had a significant impact on the results of TST by increasing the number of false negatives, but the impact on T-SPOT was relatively small (25). Our study further shows that the T-SPOT.TB positivity rate decreased as the dose of glucocorticoids increased, that is, there was a dose-effect relationship between the two. A cohort study by S. C. Yang et al. found that the risk of ATB increased with an increasing dose of glucocorticoids (26). Thus, when SLE patients receiving high-dose glucocorticoids show negative T-SPOT.TB results, probable false negatives should be suspected.

A meta-analysis showed that among 3,197 patients with rheumatic immune diseases, 71.5% of those receiving immunosuppressants had a lower possibility of positive IGRAs (OR, 0.66; 95% CI, 0.53 to 0.83) compared to those who did not receive immunosuppressive therapy, and the possibility was particularly low in patients with TNF-α antagonist treatment (OR, 0.50; 95% CI, 0.29 to 0.88) (27). Our study found that SLE patients treated with the immunosuppressants LEF and FK506 were more likely to have negative T-SPOT.TB results. LEF impedes the synthesis of pyrimidine nucleotides by inhibiting the activity of dihydrolactate dehydrogenase; on the other hand, the drug impedes tyrosine phosphorylation and the initiation of interleukin 2 (IL-2) transcription, thereby inhibiting IL-2 production, blocking the proliferation of activated lymphocytes, and reducing the production of autoantibodies (28). A Swedish case-control study by J. K. Sundbaum et al. showed that LEF could increase the risk of ATB in rheumatoid arthritis patients (adjusted OR [aOR], 6.0; 95% CI, 1.5 to 24.7) (29). FK506, a calcineurin inhibitor, can effectively inhibit the production of IL-2, IL-4, and IL-3; furthermore, it can inhibit the proliferation and the activation of T cells (30). A prospective cohort study of psoriasis patients in Taiwan showed that patients treated with FK506 had a higher risk of tuberculosis (hazard ratio [HR], 5.31; 95% CI, 1.66 to 17.01) (31). It is evident that LEF and FK506 will significantly increase the risk of ATB in patients with autoimmune diseases, while they tend to produce negative T-SPOT.TB results. Therefore, T-SPOT.TB results should be cautiously interpreted for SLE patients receiving LEF and FK506.

In summary, high disease activity, high-dose glucocorticoids, LEF, and FK506 are associated with a higher risk of ATB, as well as a higher possibility of false-negative T-SPOT.TB. Thus, for SLE patients with the above conditions, T-SPOT.TB results should be cautiously interpreted; accordingly, a positive T-SPOT.TB assay provides more convincing evidence for LTBI. In the future, tuberculosis risk stratification of SLE patients based on T-SPOT.TB results and clinical indicators can be considered.

### Limitations.

This study has some limitations. First, about 1/4 of the SLE patients did not undergo the T-SPOT.TB assay, which might have led to selection bias. However, the baseline data of patients with and without T-SPOT.TB appear comparable, so the results are still reliable. Second, with a view to feasibility, we chose the T-SPOT.TB assay for detection of LTBI without combining the QuantiFERON-TB Gold in-tube (QFT-GIT) test and TST at the same time. Although this choice was made with consideration for the accuracy of detecting LTBI in SLE patients, the prevalence of LTBI might still have been underestimated.

### Conclusions.

This study found that the prevalence of LTBI in SLE patients was about 15%, lower than the nationwide epidemiological estimate. Given the possibility of false negatives in SLE patients with T-SPOT.TB for LTBI screening, especially in those with high disease activity, high-dose glucocorticoids, and treatment with LEF and tacrolimus, attention should be paid when evaluating the presence of LTBI. We further suggest that future studies should explore tuberculosis risk stratification of SLE patients based on T-SPOT.TB results and clinical indicators.

## MATERIALS AND METHODS

### Study population.

Outpatients and inpatients with SLE who accepted T-SPOT.TB screening at 13 tertiary hospitals in eastern, central, and western China between September 2014 and March 2016 were enrolled. The inclusion criteria were (i) age over 15 years old and (ii) diagnosis of SLE according to the 1997 classification criteria of the American College of Rheumatology (ACR). The exclusion criteria were (i) pregnancy and (ii) diagnosis of ATB according to the latest Chinese standard of diagnosis for pulmonary tuberculosis (WS288-2017). This study was reviewed and approved by the ethics committees of 13 hospitals, including the Peking Union Medical College Hospital, Chinese Academy of Medical Sciences, and all subjects signed informed consent forms.

### Sampling.

The subjects in this study were obtained from the Epidemiological Study and Therapeutic Evaluation of Rheumatic Patients with Tuberculosis (ETHERTB). ETHERTB is a multicenter study based on tertiary hospitals using stratified multistage cluster sampling. The details of the sampling process have been published (32). First, China was divided into three regions based on geographic location and economic status, namely, the eastern, central, and western areas. According to the population size in the different regions, we used a simple random sampling method to select 6 out of 9, 3 out of 10, and 4 out of 12 provinces, municipalities, or autonomous regions from the eastern, central, and western regions, respectively. Second, for each selected province, municipality, or autonomous region, one tertiary general hospital was randomly chosen as the study site. Finally, eligible outpatients and inpatients with rheumatic diseases were consecutively screened and enrolled, including patients with SLE. Patients with SLE in ETHERTB who accepted T-SPOT.TB screening were enrolled in this study.

### Factors that may affect the T-SPOT.TB assay.

Gender, age, BMI, course of disease, disease activity score (according to the clinical SLEDAI-2K), dose of glucocorticoids used, use of immunosuppressants, and evidence of previous tuberculosis were included in the analyses.

### T-SPOT.TB assay.

The T-SPOT.TB kit (Oxford Immunotec, Oxford, United Kingdom) was used according to the instructions. With an EliSpot plate reader (iSpot Reader Spectrum, AID, Germany), the result was considered positive if the spot number of any well was ≥6 greater than that of blank control and was greater than or equal to two times that of blank control; it was considered inconclusive if the spot number of the blank control was greater than 10, or the spot number of the positive control was less than 20. We conducted standardized training about laboratory procedures across multiple centers and provided on-site supervision to ensure the quality of the reagents and the accuracy of the T-SPOT.TB results prior to the start of the project.

### Statistical analyses.

Statistical analyses were performed using SPSS software (IBM SPSS Statistics for Windows version 26.0). The Kolmogorov-Smirnov test was used to check normality. Normal measurement data were expressed as the mean ± standard deviation (SD), and the *t* test was applied. Non-normal measurement data were expressed as the median (interquartile range [IQR]), and the Mann-Whitney U test was applied. Counting data were expressed as percentages, and categorical data were compared using the chi-square test or Fisher’s test. Univariate and multivariable logistic regression (LR) analyses were used to determine factors affecting the T-SPOT.TB results. The univariate analysis included gender, age, BMI, course of disease, evidence of previous TB, SLEDAI-2K score, dose of glucocorticoids, and type of immunosuppressants. Significant variables from the univariate analysis were included in a multivariable logistic regression model (backward LR; entry, 0.05; removal, 0.10). A *P* value of <0.05 was considered statistically significant.
