# Supplementary material for: Latent Tuberculosis Infection and Associated Factors in Patients with Systemic Lupus Erythematosus: a Multicenter, Cross-Sectional Study
Source: Microbiol Spectr. 2023 May 9;11(3):e00848-23. doi: 10.1128/spectrum.00848-23 (PMC10269486; doi:10.1128/spectrum.00848-23)
Supplement: Supplemental file 1 — Supplemental material. Download spectrum.00848-23-s0001.doc, DOC file, 0.05 MB [file spectrum.00848-23-s0001.doc]

**The Supplementary data**

| Table S1. The frequencies of ESAT-6 and CFP-10 specific IFN-γ secreting T cells | | | | | | |
| --- | --- | --- | --- | --- | --- | --- |
|  | ESAT-6 | P value | CFP-10 | P value | Total | P value |
| Age |  | 0.141 |  | 0.037 |  | 0.027 |
| 16~40 | 40[21,119] |  | 40[17,95] |  | 84[44,196] |  |
| >40 | 52[24,118] |  | 56[24,130] |  | 112[52,266] |  |
| Evidence of previous tuberculosis | | 0.433 |  | 0.513 |  | 0.212 |
| No | 44[24,116] |  | 40[24,104] |  | 100[47,229] |  |
| Yes | 72[24,160] |  | 48[20,264] |  | 108[68,420] |  |
| SLEDAI-2K | | 0.198 |  | 0.029 |  | 0.055 |
| 0~9 | 48[24,123] |  | 46[24,120] |  | 104[48,250] |  |
| >=10 | 40[10,96] |  | 28[4,76] |  | 76[38,186] |  |
| Dose of glucocorticoids (mg/d) | | 0.614 |  | 0.047 |  | 0.159 |
| <60 | 48[24,120] |  | 44[24,116] |  | 100[48,246] |  |
| >=60 | 56[20,116] |  | 32[8,60] |  | 84[36,172] |  |
| Immunosuppressant |  |  |  |  |  |  |
| LEF |  | 0.289 |  | 0.273 |  | 0.940 |
| No | 44[24,120] |  | 48[24,110] |  | 100[48,234] |  |
| Yes | 74[28,109] |  | 32[2,59] |  | 102[49,244] |  |
| FK506 |  | 0.300 |  | 0.790 |  | 0.525 |
| No | 48[24,120] |  | 44[24,108] |  | 100[48,236] |  |
| Yes | 26[3,122] |  | 36[6,209] |  | 76[31,286] |  |
|  |  |  |  |  |  |  |
|  |  |  |  |  |  |  |
|  |  |  |  |  |  |  |
